# Parametric color-coding-derived microvascular transit time may predict infarction and reveals microcirculatory benefits of Tenecteplase in acute ischemic stroke

**DOI:** 10.3389/fneur.2026.1832149

**Published:** 2026-06-19

**Authors:** Ludwig Singer, Jeannette Becker, Maximilian Sprügel, Niklas Keller, Alexander Sekita, Jochen Sembill, Hannes Lücking, Sherif A. Mohamed, Stefan Schwab, Stefan T. Gerner, Arnd Doerfler, Tobias Engelhorn

**Affiliations:** 1 Institute of Neuroradiology, University Hospital Erlangen, Friedrich-Alexander-University Erlangen-Nürnberg, Erlangen, Germany; 2 Department of Neurology, University Hospital Erlangen, Friedrich-Alexander-University Erlangen-Nürnberg, Erlangen, Germany

**Keywords:** endovascular stroke therapy, iFLOW, parametric color coding, stroke, Tenecteplase

## Abstract

**Background:**

Despite successful macrovascular recanalization a subset of patients with a large vessel occlusion (LVO) have persisting microvascular occlusion, known as the” no-reflow-phenomenon.” In this study, we evaluated whether microvascular transit time (mTT), derived from parametric color-coding (PCC) of digital subtraction angiography (DSA) images, can serve as a tool to quantify impaired cerebral microcirculation and to assess its association with infarction. In a second step, we evaluated whether Tenecteplase (TNK), the new primary thrombolytic agent, improves microcirculatory flow compared to Alteplase (rtPA).

**Methods:**

We retrospectively analyzed 160 (110 received rtPA; 50 received TNK) presenting with anterior circulation LVO who achieved successful macrovascular recanalization (defined as TICI ≥2b). Microvascular flow was quantified using syngo iFLOW software where mTT was defined as the time from peak opacification between cortical regions and the superior sagittal sinus. Patients were stratified based on infarction presence (infarction vs. no infarction) and thrombolytic treatment (TNK vs. rtPA).

**Results:**

Prolonged mTT was significantly associated with infarction compared to non-infarcted regions (3.01 s; IQR: 2.40–3.46) vs. 2.58 s (IQR: 2.13–3.01, *p* = 0.002). An mTT threshold of >2.857 s was identified as the optimal cutoff for predicting infarction. When stratified by treatment, patients receiving TNK had significantly shorter mean mTT than those treated with rtPA, both in the infarct group (TNK: 2.57 s; IQR 2.10–3.04 vs. rtPA: 3.02 s; IQR: 2.49–3.63, *p* = 0.034) and the no-infarct group (TNK: 2.22 s; IQR: 1.87–2.74 vs. rtPA: 2.84 s; IQR: 2.4–3.27, *p* < 0.001).

**Conclusion:**

Intra-procedural flow analysis using mTT performed directly in the angio suite may be a predictor of subsequent infarction following EVT. Moreover, Tenecteplase is associated with superior microvascular circulation times compared to Alteplase, supporting its potential benefit as a preferred thrombolytic agent in acute ischemic stroke.

## 1 Introduction

Acute ischemic stroke (AIS) is a leading cause of mortality and long-term disability worldwide (1). Intravenous thrombolysis (IVT) remains the standard first-line therapy and is administered in the majority of eligible patients, often prior to endovascular thrombectomy (EVT) for large vessel occlusion (LVO) (2–4). For decades, recombinant tissue plasminogen activator (rtPA) has been the main agent used for IVT, but Tenecteplase (TNK), a genetically modified variant of rtPA, has recently emerged as a promising alternative. Due to its greater fibrin specificity, longer half-life, and single-bolus administration, TNK offers both pharmacological and logistical advantages (5, 6). This has been supported by randomized controlled trials demonstrating non-inferiority of TNK compared to rtPA in the early time window (7–11), with recent meta-analyses showing that TNK was associated with higher rates of excellent functional outcome (mRS 0–1), while rates of good functional outcome (mRS 0–2) remained comparable (12). However, its impact on cerebral microcirculation remains inadequately understood.

Conventional imaging focus primarily on large-vessel patency and often fail to capture microcirculatory dynamics. The Thrombolysis in Cerebral Infarction (TICI) grading system, which is the standard measure of procedural success, reflects macrovascular recanalization only and is not designed to capture microvascular reperfusion (13–15). The so-called ‘no-reflow phenomenon’ describes the failure of capillary reperfusion despite successful reopening of the primary vessel. Proposed mechanisms include microthrombi, endothelial swelling, pericyte-mediated constriction, and distal embolization, all of which may contribute to secondary infarct growth even after technically successful EVT (16–18). Parametric color-coding (PCC) of digital subtraction angiography (DSA) has emerged as a novel method to quantify cerebral microvascular flow in real time directly in the angio-suite. One such parameter, microvascular transit time (mTT), may serve as a surrogate for microcirculatory integrity and has been linked to functional outcomes or development of malignant edema after stroke (19, 20).

In this study, we aimed to address two primary objectives: (1) to evaluate whether impaired cerebral microcirculation, measured by mTT using PCC, is associated with infarct development despite successful LVO recanalization, and (2) to assess whether treatment with TNK results in improved microvascular reperfusion compared to rtPA.

## 2 Methods

The Stroke Research Consortium in Northern Bavaria (STAMINA; www.clinicaltrials.gov; NCT04357899; Registration No 62-21B) database is comprised of more than 5,000 patients treated for acute ischemic stroke at the University Hospital of Erlangen since 2006. Patients were screened consecutively between 2015 and 2024, with rtPA patients identified retrospectively from the STAMINA registry prior to the institutional transition to TNK, which was adopted as the primary thrombolytic agent at our center in June 2024, and TNK patients identified prospectively from June 2024 onward. Patients were eligible if they presented with a large vessel occlusion of the anterior circulation (terminal internal carotid artery [ICA] or middle cerebral artery [MCA] segments M1 or dominant M2), had an Alberta Stroke Program Early CT Score (ASPECTS) ≥ 6, and underwent successful mechanical thrombectomy defined as a Thrombolysis in Cerebral Infarction (TICI) score ≥2b. These criteria are consistent with current European Stroke Organisation (ESO) guidelines for patient selection in EVT (21). Additionally, the post-recanalization DSA run had to be of adequate quality for parametric color-coding analysis and served as a prerequisite for inclusion. No imputation was performed for missing variables. A flow chart detailing the workflow has been added into Supplementary material.

### 2.1 Image acquisition

The DSA was performed on a monoplane or biplane flat-panel detector angiography system (Axiom Artis dBA, Axiom Artis zeego, Axiom Artis Pheno or Axiom Artis Icono, Siemens Healthineers, Forchheim, Germany). All patients underwent endovascular thrombectomy under general anesthesia and a standard transfemoral approach was used. Image acquisition was performed using a 6F long sheath positioned in the cervical or subpetrous portion of the ICA. The 2D DSA series were acquired in posterior–anterior (PA) and lateral projection with a rate of 4 frames per second, as is routine in our department. For image acquisition, 8 mL of diluted contrast material (Imeron 300, Bracco Imaging, Konstanz, Germany) was injected in all series manually by an experienced operator at a flow rate of approximately 4 mL/s for 2 s validated by Gölitz et al. (22).

### 2.2 Image analysis and parametric color coding

For standardized assessment, the apical middle cerebral artery (MCA) territory was divided into three equal thirds, analogous to the apical regions of the Alberta Stroke Program Early CT Score (ASPECTS) regions (M4–M6). These apical MCA regions were then evaluated on follow-up non-contrast CT scans for the presence or absence of infarction by two blinded readers (L. S. and J. B.). Angiographic data was transferred to a Leonardo workstation (Siemens Healthineers, Forchheim, Germany) and analyzed using commercially available iFlow software (syngo iFlow, Siemens Healthineers, Forchheim, Germany). This software overlays all frames of the 2D digital subtraction angiography (DSA) series into a single parametric color-coded image, where the time to maximum opacification is visually represented. Flow analysis was performed on both posterior–anterior (PA) and lateral (LAT) projections of the post-recanalization DSA run. A point of interest (POI) was placed in the superior sagittal sinus approximately 2 cm above the confluence of sinuses. Additionally, three Regions of interest (ROIs) of approximately 720 mm^2^ each were positioned to represent the cortical areas corresponding to the apical ASPECTS regions (M4–M6) on the lateral DSA run, allowing direct comparison with the infarction zones on follow-up CT. Readers were blinded during assessment.

Only patients with LVO of the anterior circulation were included as these locations allow optimal visualization of the supplied cortical territories on the lateral DSA projection. Following the method described by Gölitz et al. (22) and Greitz (23), parameters of cerebral circulation were calculated. MTT, the primary parameter of interest, was defined as the time difference between the TTP of each cortical ROI and the superior sagittal sinus.

### 2.3 Statistical analysis

Continuous variables were tested for normal distribution using the Shapiro–Wilk test. Data is given as median with interquartile range (IQR) and compared using *t*-test or Mann–Whitney *U* test when applicable. Categorical variables were compared using the *χ*^2^ test. Receiver operating characteristic (ROC) curve analysis and Youden index were performed to determine optimal cutoff values for mTT in predicting infarction, with area under the curve (AUC) reported. Additional ROC Analysis was performed to determine whether NIHSS at admission or mean mTT can predict good functional outcome defined as NIHSS ≤ 4 at discharge. DeLong test was used to compare ROC-curves. Multivariable logistic regression models were constructed to identify independent predictors of prolonged mTT, including clinical and angiographic covariates. Prior to modeling construction, variance inflation factors (VIF) were calculated for all covariates to assess multicollinearity, with VIF below 5 considered as absence of multicollinearity. To account for baseline imbalance, inverse probability of treatment weighting (IPTW) using stabilized, trimmed weights was applied to evaluate the average treatment effect of TNK on mean mTT and infarct volume. A *p*-value < 0.05 was considered statistically significant. Statistical analyses were performed using Python version 3.12.10 (24).

## 3 Results

### 3.1 Patient characteristics

We evaluated a total of 160 patients (male: 70; female: 90) with acute ischemic stroke due to anterior circulation large vessel occlusion. The median age was 76 years (IQR: 67–82). The majority of occlusions were located in the M1 segment of the middle cerebral artery (*n* = 125), 10 occlusion of the internal carotid artery (*n* = 10) and 25 Occlusions of the middle cerebral artery in segment M2 (*n* = 25). Patients were treated with either Alteplase (rtPA, *n* = 110) or Tenecteplase (TNK, *n* = 50) prior to thrombectomy. Distribution of thrombolysis in cerebral ischemia (TICI) grades distribution was comparable between groups (rtPA vs. TNK: eTICI 2b 10% vs. 10%, eTICI 2c 14.5% vs. 24%, eTICI 3 75.5% vs. 66%; *p* = 0.336). The median number of recanalization passes was comparable between the groups (rtPA: 2.0 [IQR 1.0–3.0] vs. TNK: 1.0 [IQR 1.0–2.0]; *p* = 0.190) (Table 1).

**Table 1:** Baseline characteristics by treatment group.

Characteristic	rtPA (*n* = 110)	TNK (*n* = 50)	*p*-value
Age (years)	75.00 [67.00–81.00]	78.00 [68.50–84.00]	0.2044
Sex [female; *n* (%)]	63 (57%)	27 (54%)	0.8299
Pre-stroke mRS	0.00 [0.00–2.00]	0.00 [0.00–3.00]	0.9829
NIHSS on admission	16.00 [12.25–19.00]	15.00 [12.00–18.00]	0.3001
NIHSS at discharge	6.00 [3.75–12.00]	2.00 [1.00–4.50]	<0.0001
Door-to-Needle (min)	34.00 [23.00–48.50]	22.00 [16.00–33.00]	0.0033
Door-to-Recanalization (min)	150.00 [112.00–232.00]	106.00 [87.50–121.00]	<0.0001
TICI score; *n* (%)			0.3363
2b	11 (10%)	5 (10%)	
2c	16 (14.5%)	12 (24%)	
3	83 (75.5%)	33 (66%)	
Number of passes	2.0 [1.0–3.0]	1.0 [1.0–2.0]	0.1899

mRS: Modified Rankin Scale; NIHSS: National Institutes of Health Stroke Scale.

Baseline characteristics of patients treated with Alteplase (rtPA) or Tenecteplase (TNK). Values are presented as median [interquartile range] or counts, as appropriate. Groups were comparable in age, sex distribution, pre-stroke mRS, and admission NIHSS. Patients treated with TNK showed significantly lower NIHSS at discharge, shorter door-to-needle times, and shorter door-to-recanalization times compared to the rtPA group.

### 3.2 Procedural and angiographic flow analysis

**Figure 1:**
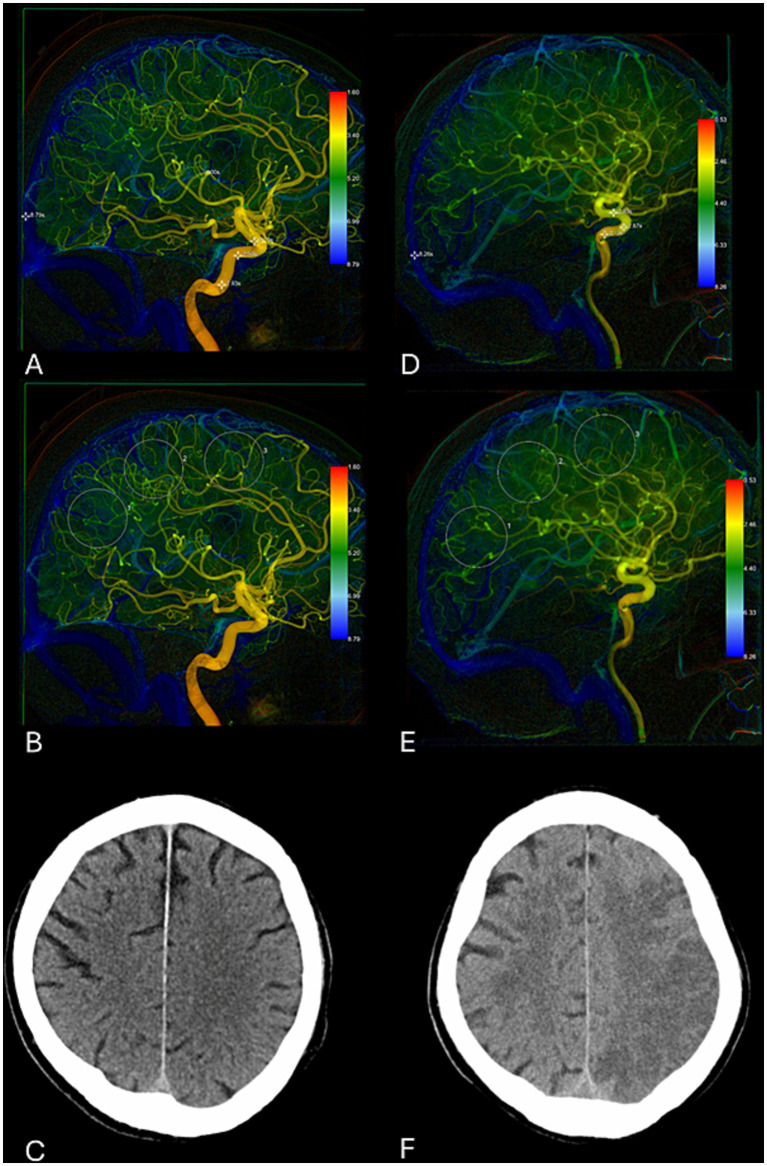
**(A,B)** Color-coded image from a lateral 2D DSA series of a 63-year-old patient shows arterial POI placement at the carotid artery and venous POI placement at the superior sagittal sinus, positioned 2 cm anterior to the confluence of sinuses. In this patient, who was treated with TNK and developed minor infarction mTT was calculated to be 2.66 s. **(C)** Non-contrast CT of the same patient within 24 h after thrombectomy without significant infarction. **(D,E)** A color-coded image from a lateral 2D DSA series of a 72-year-old patient treated with rtPA. Analogous to figure POIs and ROIs were placed. MTT was calculated to be 3.72 s and the patient subsequently developed large infarction. **(F)** Non-contrast CT showing extensive infarction involving the left hemisphere.

**Figure 2:**
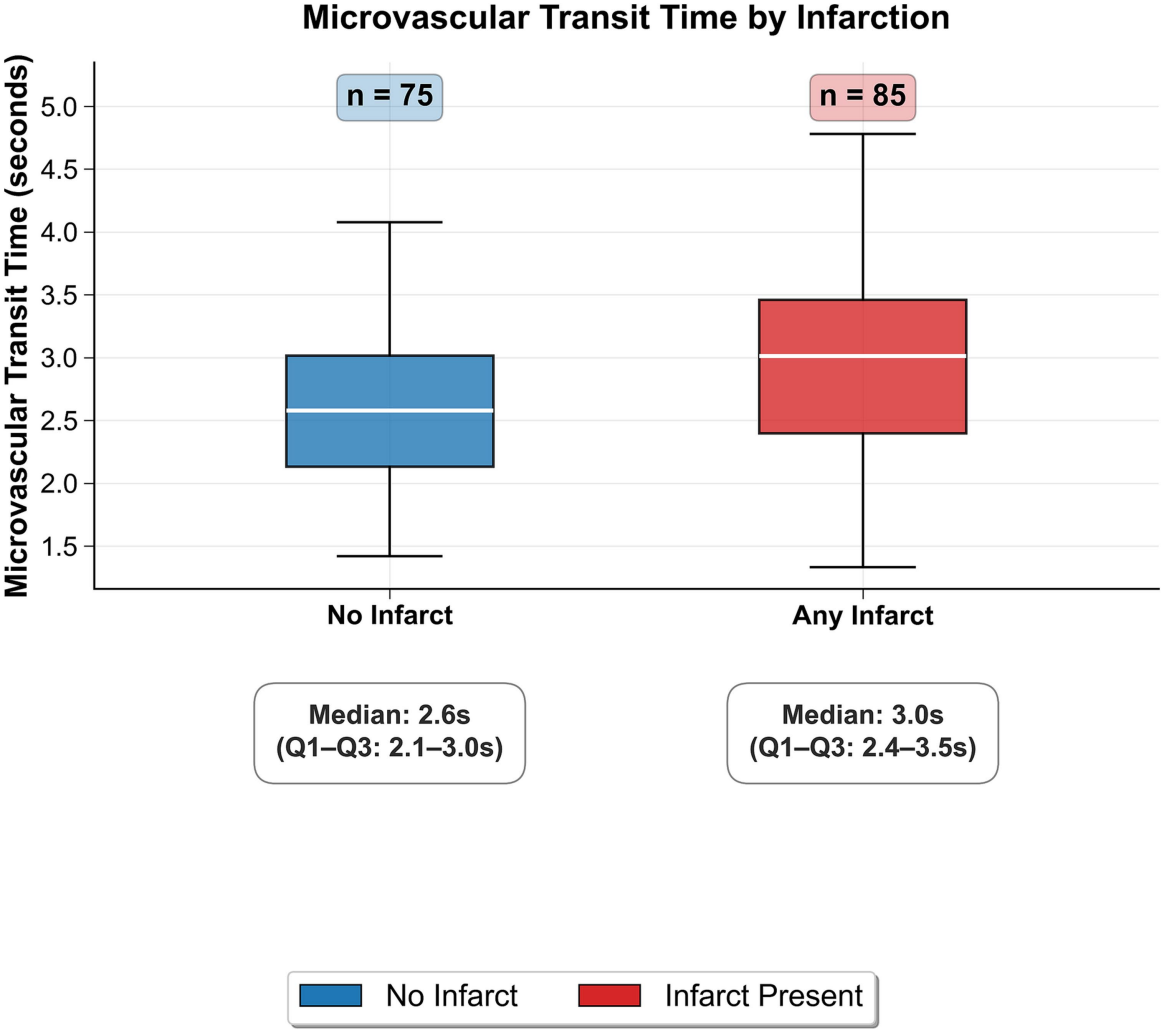
Comparison of mean microvascular transit time (mTT) between patients with and without infarction. Patients with any infarct (*n* = 85, red) showed significantly longer mTT (median 3.0 s, IQR 2.4–3.5 s) compared to those without infarction (*n* = 75, blue; median 2.6 s, IQR 2.1–3.0 s; *p* = 0.002).

Analysis of post-recanalization DSA data showed significantly prolonged mTT in infarcted regions compared to non-infarcted regions across all defined apical MCA sub-territories analogous to Figure 1: (i) M1 region: infarction 3.20 s (IQR 2.67–3.99) vs. non-infarcted 2.51 s (IQR 1.87–2.94), *p* < 0.001; (ii) M2 region: infarction 3.19 s (IQR 2.66–3.72) vs. non-infarcted 2.66 s (IQR 2.12–3.19), *p* < 0.001; (iii) M3 region: infarction 3.46 s (IQR 2.92–4.25) vs. non-infarcted 2.93 s (IQR 2.40–3.25), *p* < 0.001. The overall mean mTT was also significantly higher in patients with any regional infarction (3.01 s, IQR 2.40–3.46) compared to those without (2.58 s, IQR 2.13–3.01; *p* = 0.002) (Figure 1). The optimal cutoff for mTT in predicting infarction was 2.857 s (sensitivity 66.2%, specificity 65.5%, AUC 0.655) (Table 2 and Figure 2).

**Table 2:** Comparison of microvascular transit times between infarction and no infarction.

Parameter	Infarct (median [IQR])	No Infarct (median [IQR])	*p*-value	AUC	Optimal cutoff (mTT)
mTT-M1	3.20 [2.67–3.99]	2.51 [1.87–2.94]	*p* < 0.001	0.773	2.67
mTT-M2	3.19 [2.66–3.72]	2.66 [2.12–3.19]	*p* < 0.001	0.677	2.66
mTT-M3	3.46 [2.92–4.25]	2.93 [2.40–3.25]	*p* < 0.001	0.674	3.20
Median-mTT	3.01 [2.40–3.46]	2.58 [2.13–3.01]	*p* = 0.002	0.655	2.857

mTT, microvascular transit time.

Comparison of microvascular transit times (mTT) between patients with and without infarction. Median mTT values (with interquartile ranges) were significantly higher in patients with infarction across M1, M2, M3 segments, and for mean mTT. Optimal cutoff to differentiate patients at risk of infarction was a median of 2.857 s with an AUC of 0.655.

**Figure 3:**
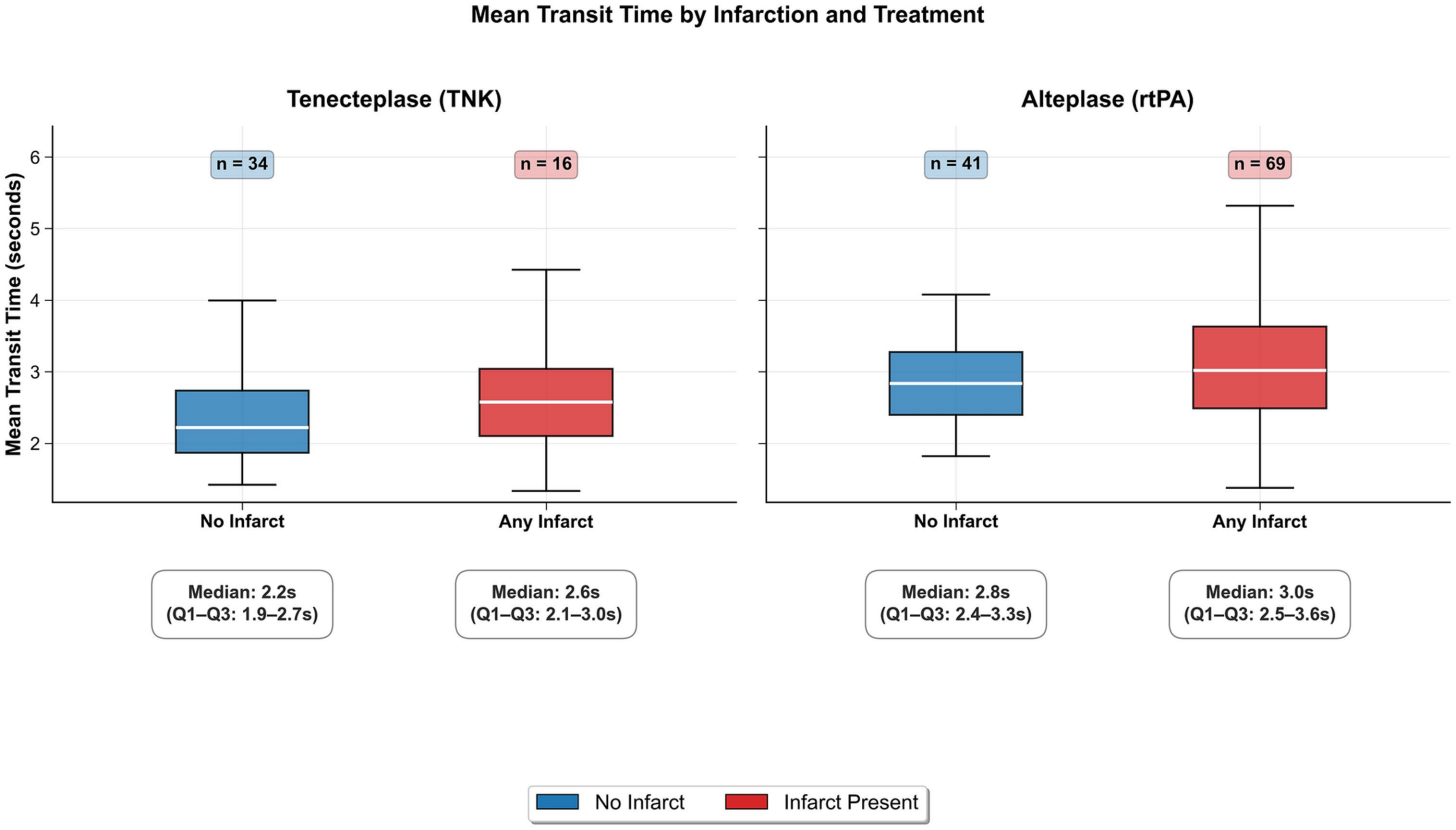
Mean microvascular transit time (mTT) stratified by infarction status and thrombolytic treatment. In the Tenecteplase (TNK) group, patients demonstrated shorter mTTs compared to those treated with rtPA. Without regional infarction, TNK patients exhibited a significantly shorter median mTT of 2.2 s (IQR 1.9–2.7 s) compared to 2.8 s (IQR 2.4–3.3 s) in the rtPA cohort (*p* < 0.001). Within infarcted regions, TNK was associated with a shorter median mTT of 2.6 s (IQR 2.1–3.0 s) compared to 3.0 s (IQR 2.5–3.6 s) for rtPA (*p* = 0.034).

When comparing thrombolytic agents, TNK was associated with significantly shorter mTTs than rtPA in the overall cohort: 2.31 s (IQR 1.89–2.83) vs. 3.01 s (IQR 2.40–3.45), *p* < 0.001. Subgroup analyses confirmed this effect: in non-infarcted regions, TNK patients had markedly lower mTT (2.22 s, IQR 1.87–2.74) compared to rtPA (2.84 s, IQR 2.40–3.27; *p* < 0.001), while in infarcted regions TNK was also associated with shorter mTT (2.57 s, IQR 2.10–3.04) compared to rtPA (3.02 s, IQR 2.49–3.63; *p* = 0.034) (Figure 3 and Table 3).

**Table 3:** Comparison of mean microvascular transit times between TNK and rtPA groups.

Subgroup/region	TNK (median [IQR])	rtPA (median [IQR])	*p*-value
Overall cohort	2.31 [1.89–2.83]	3.01 [2.40–3.45]	*p* < 0.001
No infarct	2.22 [1.87–2.74]	2.84 [2.40–3.27]	*p* < 0.001
Infarct present	2.57 [2.10–3.04]	3.02 [2.49–3.63]	*p* = 0.034

Comparison of mean microvascular transit times (mTT) between Tenecteplase (TNK) and Alteplase (rtPA) treatment groups. Across the overall cohort, as well as in patients with and without infarction, median mTT was significantly shorter in the TNK group compared to the rtPA group.

IPTW-adjusted analyses showed that after weighting, TNK remained significantly associated with shorter mean mTT in both the full model adjusting for workflow times (ATE = −0.65 s, 95% CI: −0.91 to −0.38, *p* < 0.001). The effect on infarct volume was not significant (ATE = −1.1 mL, 95% CI: −35.9 to 33.8, *p* = 0.953).

### 3.3 Clinical outcomes

Baseline stroke severity measured by NIHSS on admission was comparable between groups (median [IQR]: rtPA 16.00 [12.25–19.00] vs. TNK 15.00 [12.00–18.00]; *p* = 0.300). Pre-stroke mRS was not statistically different in both cohorts (rtPA 0.00 [0.00–2.00] vs. TNK 0.00 [0.00–3.00]; *p* = 0.983). The door-to-needle time (DTN) was shorter in the TNK cohort (22.00 [16.00–33.00] min) compared to rtPA (34.00 [23.00–48.50] min; *p* = 0.003), and door-to-recanalization (DTR) time was also reduced (TNK 106.00 [87.50–121.00] min vs. rtPA 150.00 [112.00–232.00] min; *p* < 0.001).

All variance inflation factors were below 2, indicating absence of multicollinearity among covariates included in the multivariable models. At discharge, NIHSS scores were significantly lower in the TNK group (2.00 [1.00–4.50]) compared to the previous standard of care rtPA (6.00 [3.75–12.00]; *p* < 0.001). In logistic regression, TNK use was independently associated with lower odds of prolonged mean mTT > 2.857 s (aOR = 0.10, 95% CI: 0.026–0.374; *p* = 0.001) and of mean mTT above the median (aOR = 0.13, 95% CI: 0.039–0.445; *p* = 0.001). Higher admission NIHSS was also an independent predictor of both prolonged mTT (aOR = 1.20, 95% CI: 1.054–1.360; *p* = 0.006) and mean mTT above the median (aOR = 1.13, 95% CI: 1.010–1.261; *p* = 0.033). Occlusion in the M2 segment was significantly associated with increased odds of prolonged mTT (aOR = 5.65, 95% CI: 1.147–27.781; *p* = 0.033). No other covariates, including age, pre-mRS, infarct volume, DTN or DTN-times, reached statistical significance in the regression models (*p* ≥ 0.05).

**Figure 4:**
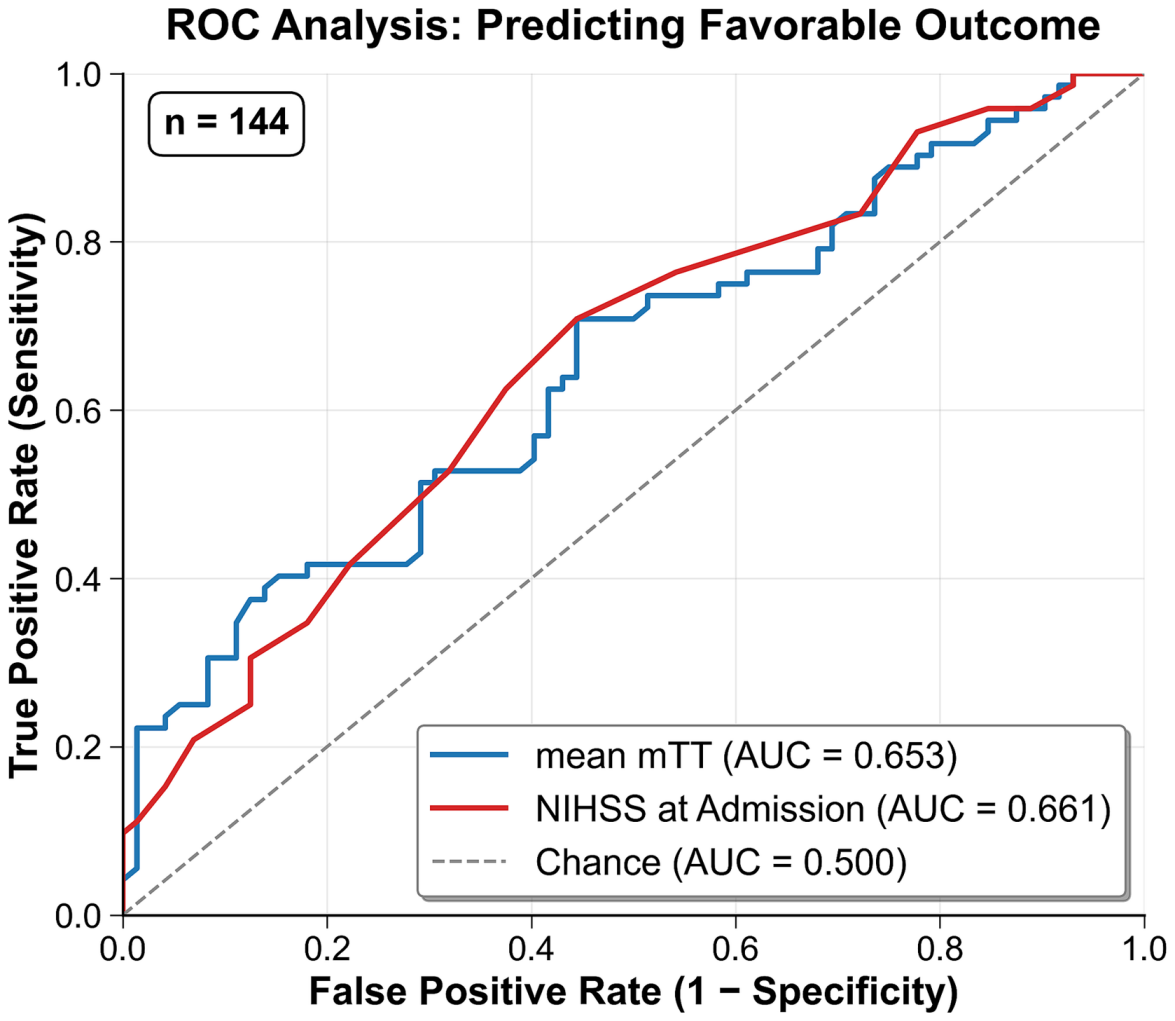
Receiver operating characteristic (ROC) analysis for predicting favorable clinical outcome (NIHSS ≤ 4 at discharge, *n* = 144). Microvascular transit time (blue) showed an area under the curve (AUC) of 0.653 with sensitivity 70.8% and specificity 55.6% at an optimal threshold of 2.857. NIHSS at admission (red) had an AUC of 0.661 with sensitivity 70.8% and specificity 55.6% at an optimal threshold of 16. The dashed line indicates chance level (AUC = 0.500).

ROC analysis showed that both NIHSS at admission to hospital (AUC = 0.661) and mean mTT (AUC = 0.653) had comparable discriminatory ability for predicting favorable outcome (discharge NIHSS ≤ 4). The difference between the two predictors was not statistically significant (*p* = 0.859) (Figure 4).

## 4 Discussion

In this study we investigated cerebral microcirculation in patients with acute ischemic stroke and successful mechanical recanalization defined as TICI 2b-3. Using parametric color-coding of digital subtraction angiographic images, we demonstrate that prolonged microvascular transit time (mTT) is significantly associated with the occurrence of infarction, and that treatment with Tenecteplase (TNK) is linked to shorter mTT values compared to Alteplase (rtPA).

These findings have several important implications. Despite complete macrovascular reperfusion, a substantial proportion of patients still develop infarction, underlining the central role of microvascular flow in tissue survival.

In our cohort, mean mTT was significantly longer in infarcted than in non-infarcted territories (3.01 s [2.40–3.46] s vs. 2.58 s [2.13–3.01], *p* = 0.002), and an mTT cutoff of 2.857 s predicted infarction with moderate accuracy. Importantly, prolonged mTT was not only associated with tissue-level outcomes but also with neurological recovery. Patients with shorter mTT values showed significantly better neurological status at discharge, with lower NIHSS scores, whereas prolonged mTT was linked to persistently higher deficits. Logistic regression confirmed that prolonged mTT was independently associated with unfavorable discharge NIHSS, even after adjustment for baseline stroke severity. Prolonged microvascular and cerebral circulation times have similarly been linked to worse functional outcome or development of malignant brain edema after thrombectomy (19, 20, 25). This underscores the potential of mTT as a clinically relevant biomarker that integrates microvascular status with functional outcome.

Our data further suggests that TNK provides superior microvascular circulation times compared to the current standard of care, rtPA. Patients treated with TNK exhibited significantly shorter mean mTT (2.31 [1.89–2.83] s vs. 3.01 [2.40–3.45] s, *p* < 0.001) and, correspondingly, lower NIHSS scores at discharge (median 2.0 [1.0–4.5] vs. 6.0 [3.8–12.0], *p* < 0.001). Notably, the association between TNK and shorter mTT remained significant after inverse probability of treatment weighting while there was no association between usage of TNK and lower infarction volume. These findings indicate that TNK’s favorable effect on microcirculation translates into a clinical benefit, beyond its pharmacological and logistical advantages such as faster door-to-needle and door-to-recanalization times. A potential biological explanation is that TNK’s greater fibrin specificity and resistance to plasminogen activator inhibitor-1 (PAI-1) allows a more effective lysis of microthrombi within peripheral vessels thereby reducing microvascular obstruction (26, 27).

The no-reflow phenomenon is certainly multifactorial, involving microthrombi, endothelial injury, pericyte constriction, and inflammation (18). Our data suggest that thrombolytic choice is a modifiable determinant of microvascular recovery. TNK’s improved fibrin selectivity may reduce microthrombus burden and enhance distal reperfusion, thereby explaining its association with lower mTT.

From a clinical perspective, mTT assessment is a quick and easy method that can be directly performed in the angio-suite after successful macrovascular recanalization without the need for further imaging. This could enable early identification of patients at risk of infarction as shown in previous analyses (28). Additionally, if mTT exceeds an identified threshold, this may indicate impaired microvascular flow, prompting immediate consideration of adjunctive therapeutic strategies such as intra-arterial lysis or calcium channel antagonists mitigating the microvascular failure (29, 30). TNK might be more suitable for this compared to rtPA due to the mentioned biological advantages and its usage after successful macrovascular recanalization have already been shown (31).

Future studies should aim to validate mTT cutoff values in larger cohorts and investigate whether PCC-guided strategies can improve clinical outcomes. Combining mTT with other imaging modalities such as intraprocedural flat-panel perfusion CT and clinical parameters could enable robust predictive models for individualized therapy (32). Additionally, integrating angiographic metrics into a multimodal framework with patient specific metrics such as local cerebral oximetry derived from advanced neuromonitoring using cerebral near-infrared spectroscopy (NIRS) could provide real-time insights into local revascularization (33).

Randomized head-to-head comparisons of TNK versus rtPA focusing specifically on microcirculatory endpoints are also needed. Current analyses such as the ANGEL-TNK trial demonstrated a greater likelihood of excellent outcomes 90 days after stroke when TNK was administered after angiographically complete recanalization (34). For cases of incomplete reperfusion, the ongoing TECNO trial (NCT05499832) will provide insights, and its results are highly anticipated.

Our findings must be interpreted in light of several limitations. This was a retrospective, single-center analysis with limited sample size, particularly in the TNK subgroup. Potential confounders such as collateral status, infarct core volume, and systemic hemodynamics including cardiac output and blood pressure during and after recanalization were not fully controlled or adjusted for. 90-day outcome data were partially not available for this retrospective cohort, therefore NIHSS at discharge was used as a surrogate measure of neurological recovery. We acknowledge that this outcome does not capture long-term functional independence. Additionally, while mTT was associated with infarction and outcome, causality cannot be established as the presence of infarction is certainly multifactorial. Furthermore, the two treatment cohorts were enrolled sequentially rather than concurrently, with rtPA patients identified retrospectively from the STAMINA registry and TNK patients enrolled from June 2024 onward. This may introduce temporal bias, as stroke workflows and periprocedural management have evolved over this period. However, the shorter door-to-needle and door-to-recanalization times observed in the TNK cohort are consistent with well-established pharmacological and logistical advantages of TNK (35). Furthermore, patients were selected as consecutive cases meeting predefined inclusion criteria specified in the methods section within each cohort, minimizing individual case selection bias. The requirement for adequate post-recanalization DSA image quality for parametric color-coding analysis may have introduced a selection bias toward patients with more complete procedural data, and the generalizability of our findings to cases with suboptimal image quality remains unknown. Further, contralateral DSA runs may be used as a reference measurement to reduce inter-individual variability in circulation times. These were not performed on a routine basis in our institution and not available for this cohort.

## 5 Conclusion

PCC-derived flow parameter mTT provides a practical and quantitative measure of cerebral microcirculation, closely linked to infarction and clinical severity. TNK improves microvascular flow compared to rtPA, reinforcing its role as the thrombolytic agent of choice in acute ischemic stroke. Incorporating mTT into the angio-suite workflow could open new opportunities for individualized treatment and identify patients at risk of the no-reflow phenomenon.

## Data Availability

The datasets analyzed during the current study are not publicly available due to patient privacy and institutional data protection regulations but are available from the corresponding author upon reasonable request. Requests to access these datasets should be directed to Ludwig.singer@uk-erlangen.de.

## Glossary

AIS: Acute Ischemic Stroke
ANGEL-TNK: Intra-arterial Thrombolysis for Acute Large Vascular Occlusion After Successful Mechanical Thrombectomy Recanalization (ANGEL-TNK)
ASPECTS: Alberta Stroke Program Early CT Score
DSA: Digital Subtraction Angiography
EVT: Endovascular Thrombectomy
ESO: European Stroke Organisation
ESMINT: European Society for Minimally Invasive Neurological Therapy
IVT: Intravenous Thrombolysis
LVO: Large Vessel Occlusion
MCA: Middle Cerebral Artery
mRS: Modified Rankin Scale
mTT: Microvascular Transit Time
NIHSS: National Institutes of Health Stroke Scale
PCC: Parametric Color Coding
POI: Point of Interest
ROI: Region of Interest
rtPA: Recombinant Tissue Plasminogen Activator (Alteplase)
STAMINA: Stroke Research Consortium in Northern Bavaria (study registry)
TICI: Thrombolysis in Cerebral Infarction (score)
TNK: Tenecteplase
TECNO: Safety and Efficacy of Intra-arterial Tenecteplase for Noncomplete Reperfusion of Intracranial Occlusions
