# Supplementary figures and images for: Parametric color-coding-derived microvascular transit time may predict infarction and reveals microcirculatory benefits of Tenecteplase in acute ischemic stroke
Source: Front Neurol. 2026 Jun 19;17:1832149. doi: 10.3389/fneur.2026.1832149 (PMC13327970; doi:10.3389/fneur.2026.1832149)

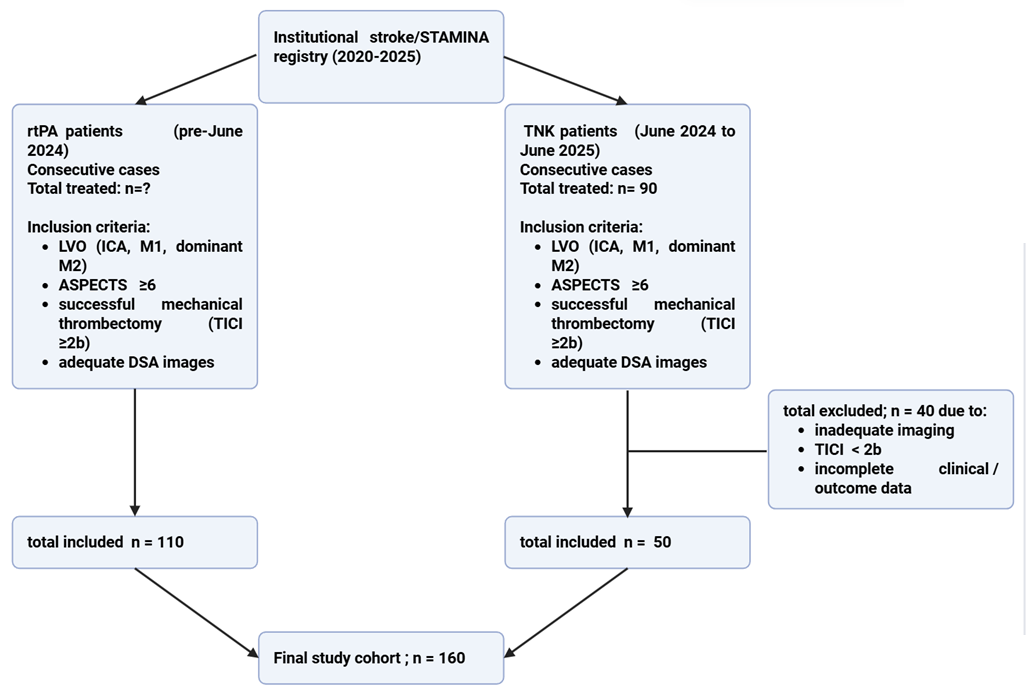

Supplement: Supplementary file 1 [file Image_1.TIF]
