# Supplementary material for: Parametric color-coding-derived microvascular transit time may predict infarction and reveals microcirculatory benefits of Tenecteplase in acute ischemic stroke
Source: Front Neurol. 2026 Jun 19;17:1832149. doi: 10.3389/fneur.2026.1832149 (PMC13327970; doi:10.3389/fneur.2026.1832149)
Supplement: Supplementary file 2 [file Supplementary_file_1.DOCX]

Supplementary Material

# Supplementary Figures


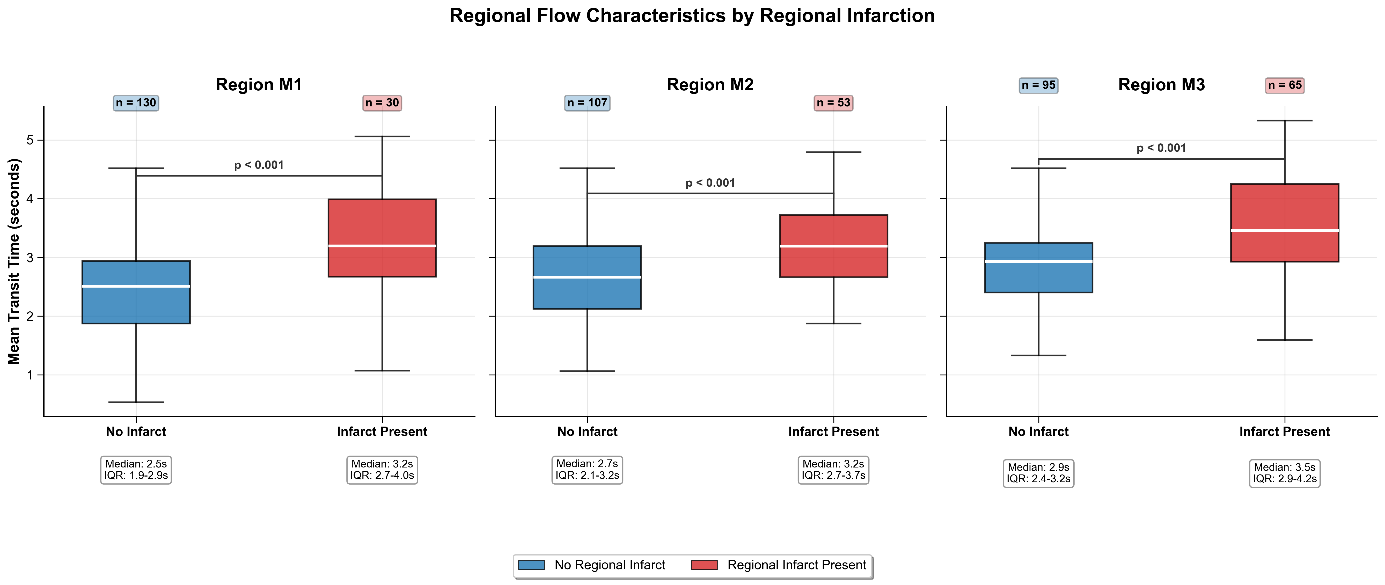


**Supplementary Figure 1**. Regional microvascular transit time (mTT) by infarction status in M1, M2, and M3 segments. Across all regions, patients with regional infarction (red) demonstrated significantly longer mTT compared to those without infarction (blue) (all p < 0.001).

# Supplementary Tables

# Supplementary Table 1: Regression results for predictors of prolonged mean mTT

| Predictor | Coefficient | 95% CI | p-value |
| --- | --- | --- | --- |
| Logistic: prolonged mean mTT (> 2.857) | | | |
| C(Occlusion)[ACI] | 0.5570 | -1.586, 2.700 | 0.610 |
| C(Occlusion)[M1] | 0.4187 | -0.225, 1.063 | 0.203 |
| C(Occlusion)[M2] | 1.7311 | 0.137, 3.325 | 0.033 |
| C(Lyse_norm)[T.tnk] | -2.3060 | -3.629, -0.983 | 0.001 |
| AGE_c | -0.0097 | -0.055, 0.035 | 0.672 |
| NIHSS_at_presentation_c | 0.1797 | 0.052, 0.307 | 0.006 |
| pre_mRS_c | 0.0434 | -0.437, 0.524 | 0.859 |
| Infarction_volume_c | 0.0067 | -0.000, 0.014 | 0.060 |
| door_to_needle_min_c | -0.0064 | -0.028, 0.016 | 0.572 |
| door_to_recan_min_c | -0.0024 | -0.010, 0.006 | 0.557 |
| Logistic: mTT above median (2.697) | | | |
| C(Occlusion)[M1] | -0.3466 | -2.137, 1.444 | 0.704 |
| C(Occlusion)[M2] | 0.9684 | -0.881, 2.818 | 0.305 |
| C(Lyse_norm)[T.tnk] | -2.0323 | -3.254, -0.811 | 0.001 |
| AGE_c | -0.0405 | -0.083, 0.002 | 0.060 |
| NIHSS_at_presentation_c | 0.1212 | 0.010, 0.232 | 0.033 |
| pre_mRS_c | 0.1808 | -0.270, 0.632 | 0.432 |
| Infarction_volume_c | 0.0052 | -0.002, 0.012 | 0.129 |
| door_to_needle_min_c | -0.0124 | -0.034, 0.009 | 0.248 |
| door_to_recan_min_c | -0.0032 | -0.011, 0.004 | 0.412 |

**Supplementary Table 1.** Logistic regression models for predictors of prolonged mean microvascular transit time (mTT). The first model used the optimal cutoff for predicting infarction (> 2.857 s), and the second applied a median split (2.697 s). In both models, treatment with Tenecteplase (TNK) was associated with significantly lower odds of prolonged mTT. Higher NIHSS on admission and M2 occlusion emerged as significant predictors, while age showed a borderline effect in the median-based model.

| **Subgroup / Region** | **TNK (median [IQR])** | **rtPA (median [IQR])** | **p-value** |
| --- | --- | --- | --- |
| Overall cohort | 2.31 [1.89–2.83] | 3.01 [2.40–3.45] | p < 0.001 |
| No infarct | 2.22 [1.87–2.74] | 2.84 [2.40–3.27] | p < 0.001 |
| Infarct present | 2.57 [2.10–3.04] | 3.02 [2.49–3.63] | p = 0.034 |
| M1 region | 2.22 [1.87–2.74] | 2.84 [2.40–3.27] | p < 0.001 |
| M2 region | 2.66 [2.12–3.19] | 3.19 [2.66–3.72] | p < 0.001 |
| M3 region | 2.93 [2.40–3.25] | 3.46 [2.92–4.25] | p < 0.001 |

**Supplementary Table 2:** Comparison of mean microvascular transit times (mTT) between Tenecteplase (TNK) and Alteplase (rtPA) groups across subgroups and vascular regions. In the overall cohort, as well as in patients with and without infarction and across M1–M3 regions, TNK treatment was consistently associated with significantly shorter mTT compared to rtPA.
